# Author Correction: Molecular basis for heat desensitization of TRPV1 ion channels

**DOI:** 10.1038/s41467-020-15691-1

**Published:** 2020-04-07

**Authors:** Lei Luo, Yunfei Wang, Bowen Li, Lizhen Xu, Peter Muiruri Kamau, Jie Zheng, Fan Yang, Shilong Yang, Ren Lai

**Affiliations:** 1 Key Laboratory of Animal Models and Human Disease Mechanisms of Chinese Academy of Sciences/Key Laboratory of Bioactive Peptides of Yunnan Province, Kunming Institute of Zoology, Kunming, Yunnan 650223 China; 2 University of Chinese Academy of Sciences, Beijing, 100049 China; 3 Key Laboratory of Medical Neurobiology, Department of Biophysics and Kidney Disease Center, First Affiliated Hospital, Institute of Neuroscience, National Health Commission and Chinese Academy of Medical Sciences, Zhejiang University School of Medicine, Hangzhou, Zhejiang 310058 China; 4 Department of Physiology and Membrane Biology, University of California, Davis, CA 95616 USA

**Keywords:** Kinetics, Molecular evolution, Ion channels in the nervous system

Correction to: *Nature Communications* 10.1038/s41467-019-09965-6, published online 13 May 2019

The original version of the Source Data file associated with this Article contained errors in the data underlying Figure 1i and Figure 5a. The HTML has been updated to include a corrected version of Source Data file.

