# Supplementary material for: Molecular basis for heat desensitization of TRPV1 ion channels
Source: Nat Commun. 2019 May 13;10:2134. doi: 10.1038/s41467-019-09965-6 (PMC6513986; doi:10.1038/s41467-019-09965-6)
Supplement: Supplementary file 1 — Supplementary Information [file 41467_2019_9965_MOESM1_ESM.pdf]

# Supplementary Information

# Molecular Basis for Heat Desensitization of TRPV1 Ion Channels

Lei Luo et al.

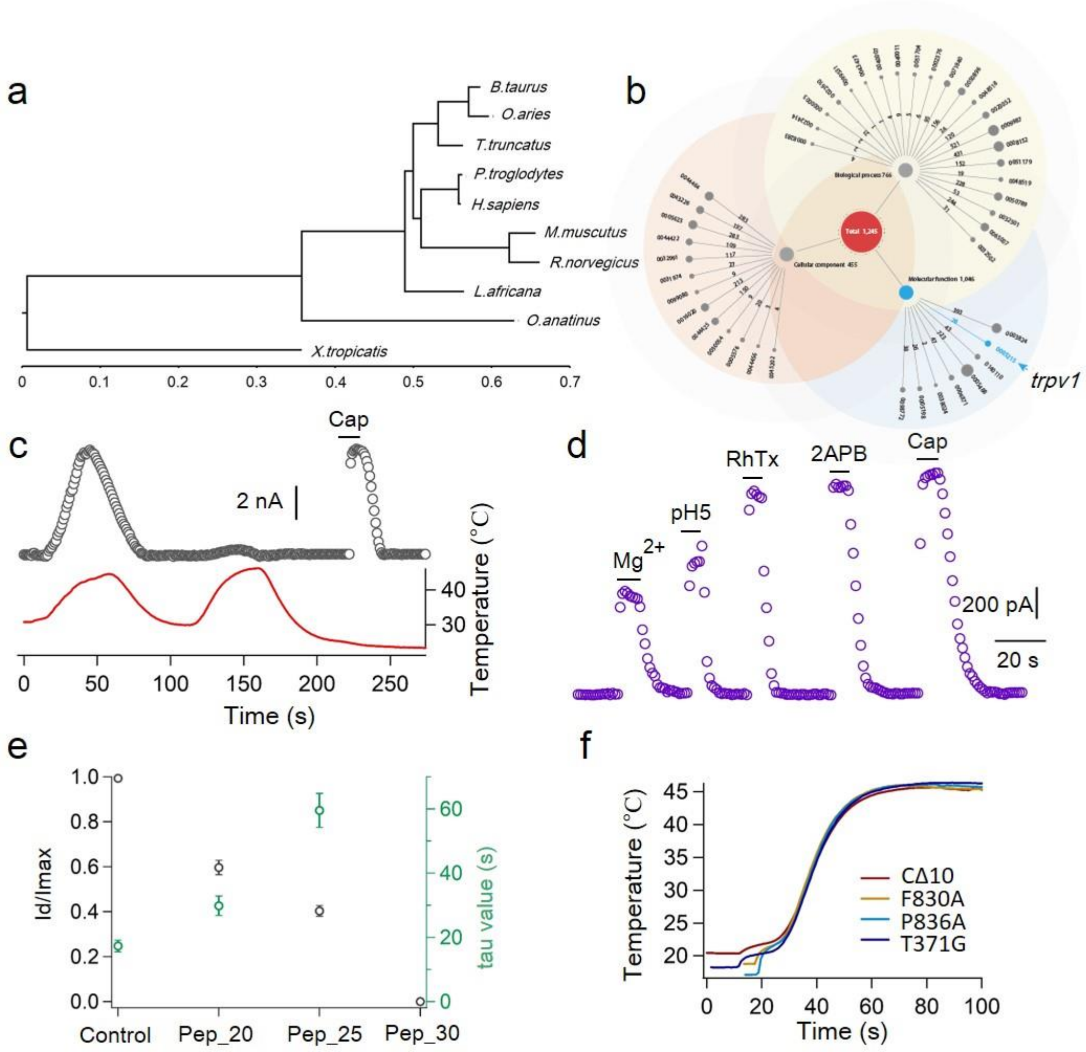


**Supplementary Figure 1.** Positive selection of platypus *trpv1* and functional conformation of platypus TRPV1 channel (pV1). **a** Consensus phylogenetic tree of species used in this study. The divergence time is labeled on the nodes. **b** Functional annotation of genes with positive selection in platypus based on PANTHER. **c** Representative current traces from HEK293T cells expressing mouse TRPV1 (mV1) evoked by repeated application of heat and supersaturated capsaicin. **d** Whole-cell pV1 currents elicited by application of $Mg^{2+}$ (130 mM), pH 5, RhTx (10 µM), 2-APB (3 mM), capsaicin (10 µM). **e** Normalized heat desensitization (Dh) degree and tau values of mV1 with C-termini peptides of different lengths. **f** The temperature ramps used in electrophysiological recordings from channel mutants, respectively. All statistical data are given as mean ± s.e.m.

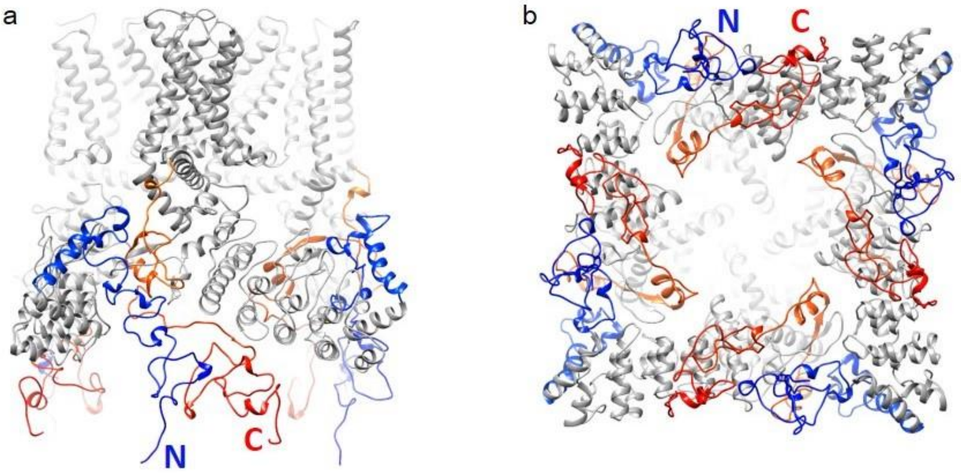


**Supplementary Figure 2.** *de novo* model of the distal N and C termini based on the cryo-EM structures. Ribbon diagram of mV1 atomic model with each of the four identical subunits color-coded, showing views from side (**a**) and bottom (**b**) focused on N and C termini.

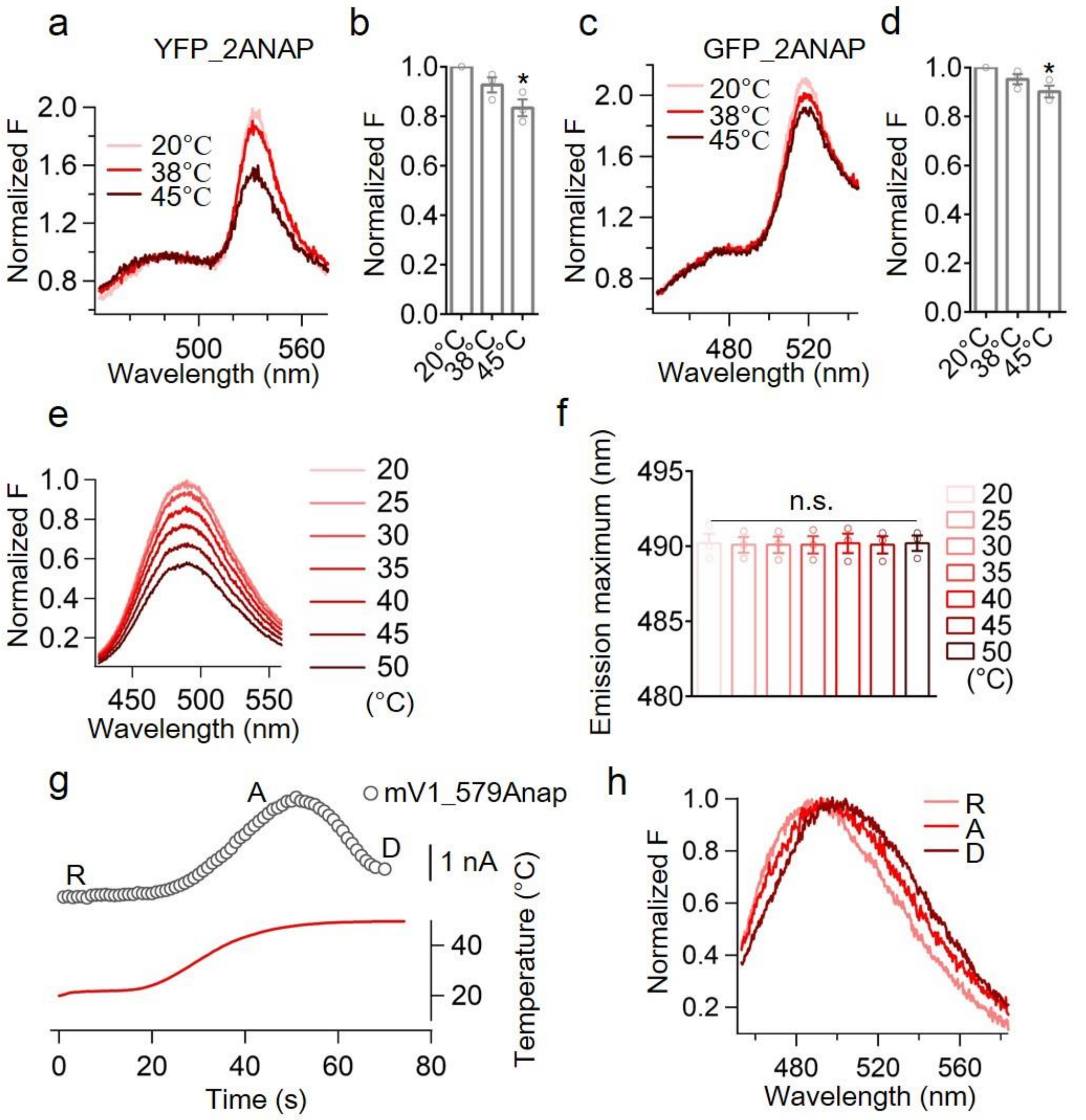


**Supplementary Figure 3.** Heat-evoked current and emission spectra of ANAP-incorporated mouse TRPV1 (mV1_579ANAP) or YFP/GFP. **a** The spectra of YFP_2ANAP were acquired at 20, 38 and 45 °C. **b** The spectra in panel **a** were normalized to the ANAP intensity (n=3, two-sided t-test: *p<0.01). **c** The spectra of GFP_2ANAP were acquired at 20, 38 and 45 °C. **d** The spectra in panel **c** were normalized to the ANAP intensity (n=3, two-sided t-test: *p<0.01). **e** The spectra of ANAP were acquired at different temperatures. **f** The emission peak of ANAP at different temperatures (n=3, two-sided t-test: n.s., not significant). **g** Representative current induced by a temperature ramp recorded from HEK 293T cells expressing mV1_579ANAP. **h** Representative emission spectra at resting (R), activated (A) and desensitized (D) states of ANAP incorporated mV1 (mV1_579ANAP). Data are mean ± s.e.m.

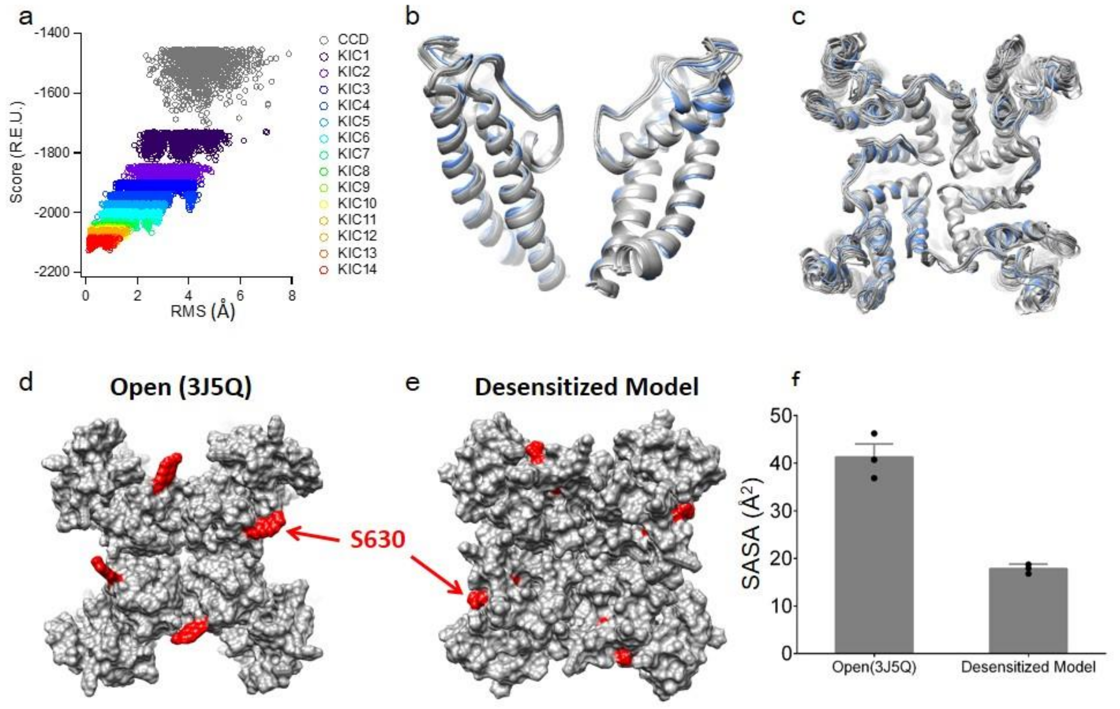


**Supplementary Figure 4.** Modeling of the TRPV1 channel in the desensitized state. **a** The models after fifteen rounds of KIC loop modeling exhibited a funnel-shaped distribution of total energy calculated by Rosetta (R.E.U., Rosetta energy unit). **b** and **c** Top five models after the fifteen round of loop modeling were well converged. The model with the lowest energy (blue) was chosen as the desensitized-state model. **d** and **e** ANAP was incorporated at the 630 sites (red) in the open (**d**) and desensitized (**e**) states, respectively. **f** SASA of ANAP at mV1_630 site was significantly decreased in the desensitized state (n=3, two-sided t-test: ***p<0.001). Data are mean ± s.e.m.

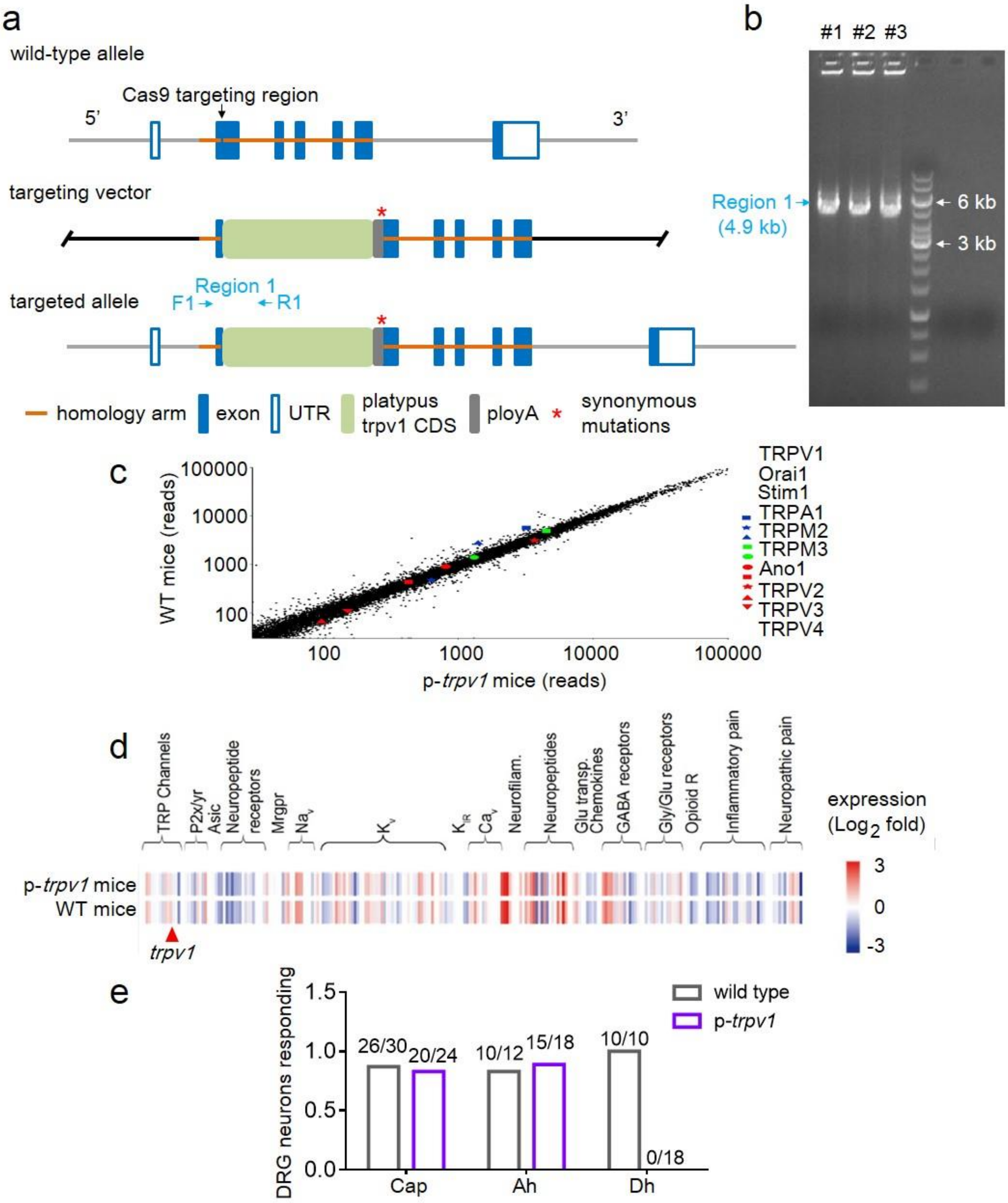

**Supplementary Figure 5.** p-*trpv1* transgenic mice construction and functional confirmation. **a** Schematic depiction of p-*trpv1* mice targeting strategy. Genomic region of mouse *trpv1* locus is diagrammed. Solid bars represent open reading frames (ORFs); open bars represent UTRs. **b** Northern blot analysis of p-*trpv1* mRNA expression in DRG from p-*trpv1* $^{+/+}$ mice. The 4.9-kb band identified with the primer pair illustrated in panel a. **c** Transcriptome-wide comparison of mRNA expression between wild-type and p-*trpv1* mice. Several ion channels that have been previously implicated in heat sensing are indicated. **d** Heat map showing the differential expression levels in wild-type and p-*trpv1* mice of a set of about 200 genes implicated in somatosensation. **e** The

percentage of wild-type and p-*trpv1* dorsal root ganglion neurons (DRGs) responding to 10 μM capsaicin, and percentages of heat-induced activation and desensitization of wild-type and p-*trpv1* DRG neurons responding to continues heat.

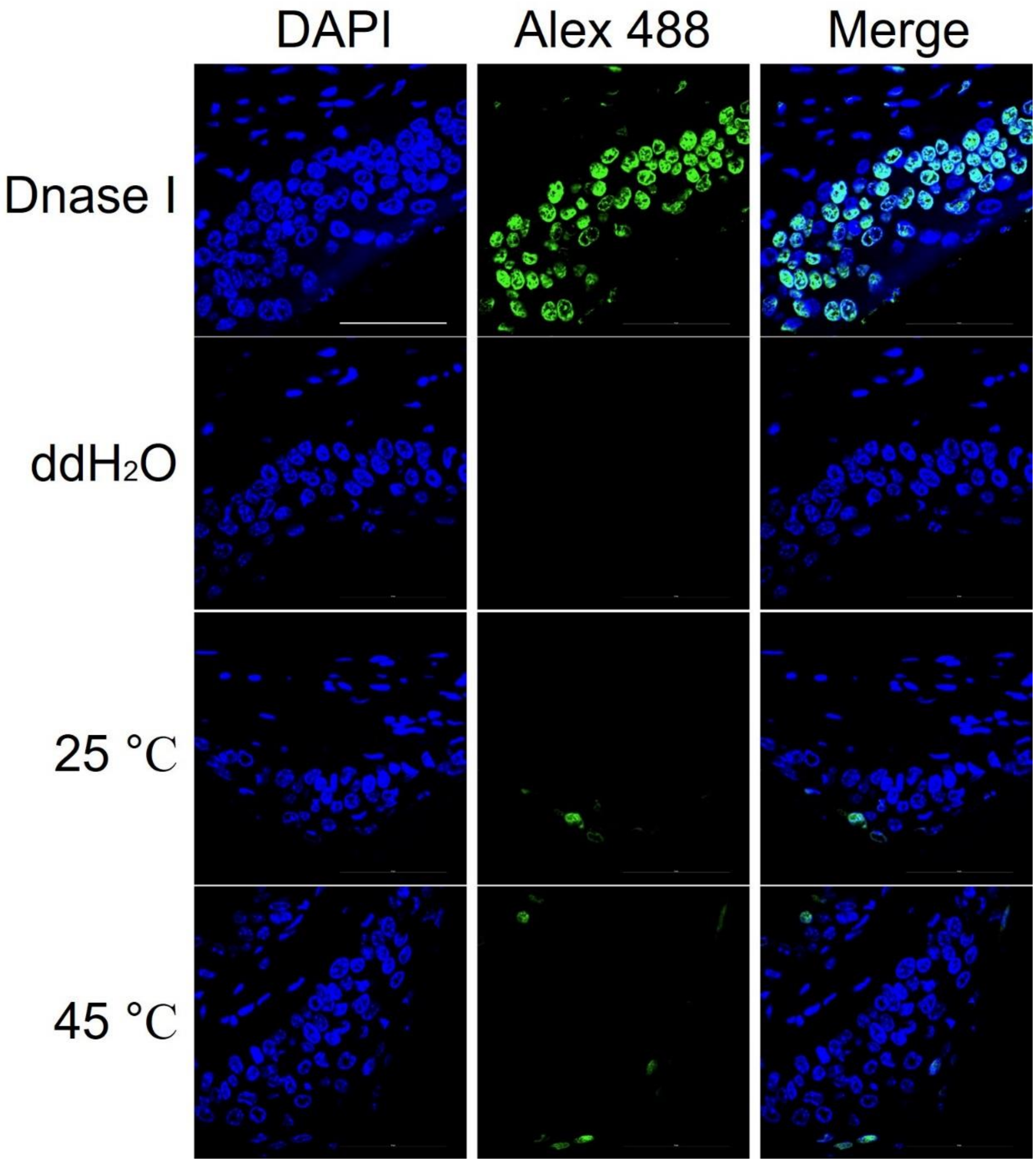


**Supplementary Figure 6.** Detection of apoptosis induced by heat using TUNEL assay. Apoptosis was detected by TUNEL staining (green fluorescence) and the nuclei were stained by DAPI (blue fluorescence) in pV1 over-expressing cells. Scale bar, 50 μm.

**Supplementary Table 1. Functional comparison of seven mammalian TRPV1 channels.**

| Species | EC50_Capsaicin (nM) | Threshold of Ah (°C) | Dh transition | τ value_Dh (s) |
|---|---|---|---|---|
| Mouse | 289 ± 17 (n = 3) | 39.8 ± 0.6 (n = 5) | Yes | 17.3 ± 0.5 (n = 3) |
| Platypus | 307 ± 23 (n = 5) | 34.0 ± 0.5 (n = 8) | No | n. d. |
| Human | 269 ± 16 (n = 3) | 40.1 ± 0.7 (n = 4) | Yes | 17.4 ± 0.5 (n = 3) |
| Fruit bat | 354 ± 25 (n = 6) | 40.2 ± 0.7 (n = 3) | Yes | 18.2 ± 0.4 (n = 3) |
| Tree shrew | 5260 ± 140 (n = 3) | 36.1 ± 0.5 (n = 4) | Yes | 19.1 ± 0.5 (n = 3) |
| Polar bear | 313 ± 21 (n = 3) | 40.6 ± 0.4 (n = 3) | Yes | 17.7 ± 0.6 (n = 3) |
| Camel | 540 ± 37 (n = 5) | 41.3 ± 0.9 (n = 6) | Yes | 20.3 ± 0.4 (n = 4) |

The Latin name of all species was shown in the method. All statistical data are expressed as means ± s.e.m. n.d. indicates no data. $EC_{50}$, effector concentration for half-maximum response; Ah, heat-induced activation; Dh, heat-induced desensitization; τ_Dh, tau value of the heat-induced desensitization.

**Supplementary Table 2. Functional comparison of mV1, pV1 and pV1's chimeras.**

| Channel name | $EC_{50}$_Capsaicin (nM) | Threshold of Ah (°C) | Dh transition | τ value_Dh (s) |
|---|---|---|---|---|
| mV1 | 299 ± 10 (n = 3) | 39.8 ± 0.5 (n = 6) | Yes | 17.3 ± 0.5 (n = 5) |
| pV1 | 301 ± 12 (n = 5) | 34.0 ± 0.4 (n = 8) | No | n. d. |
| pV1_mC | 310 ± 19 (n = 6) | 34.2 ± 0.4 (n = 5) | No | n. d. |
| pV1_mN | 313 ± 16 (n = 6) | 33.7 ± 0.4 (n = 5) | No | n. d. |
| pV1_mNC | 306 ± 13 (n = 6) | 34.0 ± 0.5 (n = 9) | Yes | 16.9 ± 0.4 (n = 5) |

All statistical data are expressed as means ± s.e.m. n.d. indicates no data. $EC_{50}$, effector concentration for half-maximum response; Ah, heat-induced activation; Dh, heat-induced desensitization; τ_Dh, tau value of the heat-induced desensitization.

**Supplementary Table 3. Functional comparison of mV1 and its truncation mutants.**

| Channel name | $EC_{50}$_Capsaicin (nM) | Threshold of Ah (°C) | Dh transition | τ value_Dh (s) |
|---|---|---|---|---|
| mV1 | 299 ± 10 (n = 3) | 39.8 ± 0.5 (n = 3) | Yes | 17.3 ± 0.5 (n = 3) |
| CΔ30 ($Glu^{747}$-$Phe^{776}$) | 372 ± 43 (n = 5) | 37.6 ± 0.6 (n = 6) | Yes | 18.8 ± 0.9 (n = 6) |
| CΔ23 ($Ser^{777}$-$Asp^{799}$) | 398 ± 51 (n = 3) | 37.8 ± 0.6 (n = 6) | Yes | 20.0 ± 0.7 (n = 6) |
| CΔ28 ($Ala^{800}$-$Ala^{827}$) | 343 ± 37 (n = 3) | 38.2 ± 0.6 (n = 6) | Yes | 20.3 ± 7 (n = 6) |
| CΔ10 ($Phe^{830}$-$Lys^{839}$) | 354 ± 39 (n = 6) | 39.0 ± 0.5 (n = 9) | No | n. d. |

**Supplementary Table 4. Functional comparison of mV1 and its point mutations.**

| Channel name | $EC_{50}$_Capsaicin (nM) | Threshold of Ah (°C) | Dh transition | τ value_Dh (s) |
|---|---|---|---|---|
| mV1 | 299 ± 10 (n = 3) | 39.8 ± 0.5 (n = 3) | Yes | 17.3 ± 0.5 (n = 3) |
| S116A | 186 ± 11 (n = 3) | 39.5 ± 0.6 (n = 3) | Yes | 18.0 ± 0.6 (n = 4) |
| T371G | 322 ± 16 (n = 3) | 39.9 ± 0.6 (n = 4) | Yes | 34.5 ± 1.9 (n = 6) |
| F830A | 338 ± 27 (n = 3) | 40.4 ± 0.5 (n = 3) | Yes | 40.0 ± 0.6 (n = 6) |
| K831A | 354 ± 30 (n = 3) | 39.9 ± 0.4 (n = 3) | Yes | 17.5 ± 0.6 (n = 3) |
| D832A | 361 ± 35 (n = 3) | 39.4 ± 0.3 (n = 3) | Yes | 17.8 ± 0.4 (n = 3) |
| S833A | 344 ± 26 (n = 3) | 39.4 ± 0.5 (n = 3) | Yes | 17.1 ± 0.5 (n = 3) |
| M834A | 329 ± 23 (n = 3) | 39.9 ± 0.4 (n = 3) | Yes | 16.8 ± 0.6 (n = 3) |
| P836A | 315 ± 24 (n = 3) | 40.1 ± 0.4 (n = 3) | Yes | 39.9 ± 0.7 (n = 6) |
| E838A | 565 ± 54 (n = 3) | 39.1 ± 0.4 (n = 3) | Yes | 17.4 ± 0.5 (n = 3) |
| K839A | 293 ± 12 (n = 3) | 39.5 ± 0.6 (n = 3) | Yes | 17.7 ± 0.4 (n = 3) |

All statistical data are expressed as means ± s.e.m. n.d. indicates no data. $EC_{50}$, effector concentration for half-maximum response; Ah, heat-induced activation; Dh, heat-induced desensitization; τ_Dh, tau value of the heat-induced desensitization.

**Supplementary Table 5. Functional confirmation of ANAP-incorporated TRPV1 channels.**

| Labeled position | $EC_{50}$_Capsaicin (nM) | low pH | 2APB | Threshold of Ah (°C) | Dh transition |
|---|---|---|---|---|---|
| mV1_241 | 290 ± 9 (n = 5) | Yes | Yes | 37.9 ± 0.4 (n = 3) | Yes |
| pV1_241 | 302 ± 11 (n = 6) | Yes | Yes | 33.9 ± 0.3 (n = 6) | No |
| mV1_575 | n. d. | n. d. | n. d. | n. d. | n. d. |
| mV1_579 | 293 ± 8 (n = 3) | Yes | Yes | 37.8 ± 0.4 (n = 3) | Yes |
| mV1_600 | n. d. | n. d. | n. d. | n. d. | n. d. |
| mV1_603 | n. d. | n. d. | n. d. | n. d. | n. d. |
| mV1_605 | n. d. | n. d. | n. d. | n. d. | n. d. |
| mV1_630 | 299 ± 10 (n = 4) | Yes | Yes | 37.9 ± 0.3 (n = 5) | Yes |
| pV1_643 | 306 ± 12 (n = 6) | Yes | Yes | 33.8 ± 0.3 (n = 7) | No |
| mV1_633 | n. d. | n. d. | n. d. | n. d. | n. d. |
| mV1_636 | n. d. | n. d. | n. d. | n. d. | n. d. |
| mV1_640 | n. d. | n. d. | n. d. | n. d. | n. d. |
| mV1_648 | 296 ± 7 (n = 6) | No | Yes | 38.2 ± 0.3 (n = 4) | Yes |
| mV1_650 | n. d. | n. d. | n. d. | n. d. | n. d. |
| mV1_651 | 288 ± 8 (n = 6) | Yes | Yes | 37.7 ± 0.4 (n = 5) | Yes |
| mV1_654 | 304 ± 10 (n = 6) | Yes | Yes | 38.1 ± 0.3 (n = 6) | Yes |
| mV1_656 | n. d. | n. d. | n. d. | n. d. | n. d. |
| mV1_672 | n. d. | n. d. | n. d. | n. d. | n. d. |
| mV1_673 | n. d. | n. d. | n. d. | n. d. | n. d. |
| mV1_676 | n. d. | n. d. | n. d. | n. d. | n. d. |
| mV1_677 | n. d. | n. d. | n. d. | n. d. | n. d. |
| mV1_680 | n. d. | n. d. | n. d. | n. d. | n. d. |
| mV1_684 | n. d. | n. d. | n. d. | n. d. | n. d. |
| mV1_685 | 295 ± 11 (n = 8) | Yes | Yes | 37.8 ± 0.4 (n = 6) | Yes |

All statistical data are expressed as means ± s.e.m. n.d. indicates no data. $EC_{50}$, effector concentration for half-maximum response; Ah, heat-induced activation; Dh, heat-induced desensitization; low pH, pH5; 2APB, 2-aminoethoxydiphenyl borate.

**Supplementary Table 6. Primers used in this study to identify the genotype of transgenic mice.**

| primer | sequence |
|---|---|
| F1 | 5’-CAGAGTGCTAGAGATGCTTTCAATC-3’ |
| R1 | 5’-TGCGTTTAATCCCTTCACTGTTC-3’ |

**Supplementary Table 7. A routine urine test in WT & p-*trpv1* mice.**

| Project | WT (n=3) | p-*trpv1* (n=3) |
|---|---|---|
| PH | 5.5 ± 0.0 | 5.5 ± 0.0 |
| Glu - (mmol/L) | 0 ± 0.0 | 0 ± 0.0 |
| Vc +3 (mmol/L) | 5.6 ± 0.13 | 5.6 ± 0.14 |
| SG | 1.030 ± 0.000 | 1.030 ± 0.007 |
| BLD - (Cell /μL) | 0 ± 0.0 | 0 ± 0.0 |
| PRO +- (g/L) | 0.15 ± 0.01 | 0.15 ± 0.00 |
| BIL - (μmol/L) | 0 ± 0.0 | 0 ± 0.0 |
| URO +1 (μmol/L) | 33 ± 0 | 33 ± 0 |
| KET +1 (mmol/L) | 1.5 ± 0.05 | 1.5 ± 0.05 |
| WBC- (Cell /μL) | 0 ± 0.0 | 0 ± 0.0 |

Data are expressed as mean ± s.e.m. from 3 mice.

**Supplementary Table 8. Routine blood test in WT & p-*trpv1* mice.**

| Project | WT (n=3) | p-*trpv1* (n=3) |
|---|---|---|
| WBC ($\times 10^9$/L) | 5.0 ± 1.92 | 12.3 ± 1.40 |
| Lymph# ($\times 10^9$/L) | 2.7 ± 1.12 | 8.1 ± 2.09 |
| Mon# ($\times 10^9$/L) | 0.2 ± 0.05 | 0.5 ± 0.12 |
| Gran# ($\times 10^9$/L) | 2.1 ± 0.28 | 3.7 ± 0.62 |
| Lymph (%) | 54.5 ± 2.49 | 66.2 ± 7.27 |
| Mon (%) | 3.1 ± 0.22 | 3.7 ± 0.49 |
| Gran (%) | 42.4 ± 6.06 | 30.1 ± 3.35 |
| RBC ($\times 10^{12}$/L) | 9.81 ± 1.02 | 11.13 ± 0.80 |
| HGB (g/L) | 148 ± 10.66 | 165 ± 13.91 |
| HCT (%) | 46.5 ± 4.02 | 54.7 ± 7.30 |
| MCV (fL) | 47.5 ± 1.27 | 49.2 ± 2.19 |
| MCH (pg) | 15.0 ± 0.74 | 14.8 ± 0.37 |
| MCHC (g/L) | 318 ± 7.76 | 301 ± 11.34 |
| RDW (%) | 14.5 ± 0.38 | 14.2 ± 0.45 |
| PLT ($\times 10^9$/L) | 464 ± 18.57 | 435 ± 17.93 |
| MPV (fL) | 5.3 ± 0.34 | 5.7 ± 0.60 |
| PDW | 15.9 ± 0.75 | 16.7 ± 0.54 |
| PCT (%) | 0.245 ± 0.004 | 0.247 ± 0.004 |

Data are expressed as mean ± s.e.m. from 3 mice.

**Supplementary Table 9. Blood bio-chemistry examination in WT & p-*trpv1* mice.**

| Project | WT (n=3) | p-*trpv1* (n=3) |
|---|---|---|
| TP (g/L) | 48.3 ± 3.7 | 55.8 ± 4.4 |
| ALB (g/L) | 33.7 ± 1.9 | 37.5 ± 2.8 |
| GLB (g/L) | 14.6 ± 2.1 | 18.3 ± 1.9 |
| ALB/GLB | 2.31 ± 0.20 | 2.05 ± 0.04 |
| ALT (IU/L) | 61.10 ± 4.80 | 80.00 ± 8.50 |
| AST (IU/L) | 254.8 ± 17.1 | 337.5 ± 33.2 |
| AST/ALT | 4.20 ± 0.08 | 4.20 ± 0.05 |
| TB (μmol/L) | 0.8 ± 0.1 | 1.3 ± 0.2 |
| DB (μmol/L) | 0.70 ± 0.05 | 0.8 ± 0.08 |
| IDBIL (μmol/L) | 0.2 ± 0.0 | 0.5 ± 0.1 |
| TBA (μmol/L) | 7.6 ± 0.4 | 6.7 ± 0.8 |
| ALP (IU/L) | 261.0 ± 30.8 | 231.5 ± 22.1 |
| Urea (mmol/L) | 9.21 ± 0.76 | 11.38 ± 0.99 |
| Cre (μmol/L) | 10.20 ± 0.80 | 13.40 ± 0.67 |
| UA (μmol/L) | 64.7 ± 9.9 | 105.3 ± 15.2 |
| GLU (mmol/L) | 7.9 ± 0.4 | 7.6 ± 0.4 |
| TC (mmol/L) | 1.88 ± 0.19 | 2.57 ± 0.40 |
| F-CHOL (mmol/L) | 0.38 ± 0.07 | 0.55 ± 0.07 |
| TG (mmol/L) | 1.05 ± 0.06 | 1.17 ± 0.13 |
| HDL-C (mmol/L) | 1.42 ± 0.20 | 1.87 ± 0.28 |
| LDL-C (mmol/L) | 0.41 ± 0.03 | 0.55 ± 0.05 |
| apoA1 (g/L) | 0.03 ± 0.00 | 0.03 ± 0.00 |
| apoB (g/L) | 0.12 ± 0.03 | 0.20 ± 0.04 |
| Ca (mmol/L) | 2.29 ± 0.15 | 2.56 ± 0.13 |

Data are expressed as mean ± s.e.m. from 3 mice.

**Supplementary Table 10. Primers used in this study to generate TRPV1 chimeras.**

| Name | Primer-Forward | Primer-Reverse |
|---|---|---|
| pV1_mNC_mV1 | ctgatgggggagacagtgaa<br>caagattgcacaagagagc | gaagttgaagtaaaatatgc<br>gcttgacaaatctgtcccac |
| pV1_mNC_pV1 | gacagatttgtcaagcgc<br>atattttacttcaacttcttc | ctcttgtgcaatcttgttca<br>ctgtctcccccatcagggc |
| pV1_mC_mV1 | ctgatgggggagacagtga<br>acaagattgcacaagagagc | ccgggcccgcggtaccgtt<br>ttctcccctggggccatgga |
| pV1_mC_pV1 | atggccccaggggagaaaa<br>cggtaccgcgggcccgggat | ctcttgtgcaatcttgttca<br>ctgtctcccccatcagggc |
| pV1_mN_mV1 | gatctcgagctcaagcttat<br>ggagaaatgggctagctta | gaagttgaagtaaaatatgc<br>gcttgacaaatctgtccca |
| pV1_mN_pV1 | gacagatttgtcaagcgc<br>atattttacttcaacttcttc | gctagcccatttctccataa<br>gcttgagctcgagatctga |
| mC_mV1 | gctagcgtttaaacttaaaa<br>caagattgcacaagagagc | ctggatatctgcagaatttt<br>tctcccctggggccatgga |
| pC_pV1 | gctagcgtttaaacttaaaa<br>caaggtctcgcaagaaagc | ctggatatctgcagaatttt<br>cttctaaaataagtgactc |

**Supplementary Table 11. Primers used in this study to generate mV1 truncation mutants.**

| Name | Primer-Forward | Primer-Reverse |
|---|---|---|
| CΔ30 ($Glu^{747}$-$Phe^{776}$) | agggtggattccctgcgg<br>tcaggccgagtttcaggg | ccgcagggaatccaccct<br>gaagcaccaccggaagtc |
| CΔ23 ($Ser^{777}$-$Asp^{799}$) | ctgagcttcgcaagcact<br>cgagataggcatagcacc | agtgcttgcgaagctcag<br>ggtgcgcttgacgccctc |
| CΔ28 ($Ala^{800}$-$Ala^{827}$) | ctgagggacgaggtcttc<br>aaggattccatggcccca | gaagacctcgtccctcag<br>aaggggaaccagggcaaa |
| CΔ10 ($Phe^{830}$-$Lys^{839}$) | gctgaggtctcagggaag<br>ccgaattctgcagtcgac | cttccctgagacctcagc<br>atcctctggcttaaggga |

**Supplementary Table 12. Primers used in this study to generate mV1 point mutations.**

| Name | Primer-Forward | Primer-Reverse |
|---|---|---|
| S116A | gatcgcagggcaatcttcgacgctgtggctcagagc | gtcgaagattgccctgcgatcatagagccttggggg |
| T371G | aggaagttcgcagaatgggcctatgggcccgtgcac | ggcccattctgcgaacttcctggacaggtgccggca |
| F830A | gctgaggtcgcaaaggattccatggccccaggggag | ggaatcctttgcgacctcagcatcctctggcttaag |
| K831A | gaggtcttcgcagattccatggccccaggggagaaa | catggaatctgcgaagacctcagcatcctctggctt |
| D832A | gtcttcaaggcatccatggccccaggggagaaatca | ggccatggatgccttgaagacctcagcatcctctgg |
| S833A | ttcaaggatgcaatggccccaggggagaaatcaggg | tggggccattgcatccttgaagacctcagcatcctc |
| M834A | aaggattccgcagccccaggggagaaatcagggaag | ccctggggctgcggaatccttgaagacctcagcatc |
| P836A | tccatggccgcaggggagaaatcagggaagccgaat | tttctcccctgcggccatggaatccttgaagacctc |
| E838A | gccccaggggcaaaatcagggaagccgaattctgca | ccctgattttgcccctggggccatggaatccttgaa |
| K839A | ccaggggaggcatcagggaagccgaattctgcagtc | cttccctgatgcctcccctggggccatggaatcctt |

**Supplementary Table 13. Primers used in this study to generate ANAP-incorporated TRPV1 channels.**

| Name | Primer-Forward | Primer-Reverse |
|---|---|---|
| mV1_241 | aagaaaacctaggggaggcctggcttctactttggt | aggcctcccctaggttttcttgaagaagtccccgtt |
| pV1_241 | aagaaaacctagggccggccgggcttttattttggt | cggccggccctaggttttcttaaagaagtcaccatg |
| mV1_575 | aagatgatctagagagacctgtgtcggtttatgttc | caggtctctctagatcatcttctcaatcatgacagc |
| mV1_579 | agagacctgtagcggtttatgttcgtctacctcgtg | cataaaccgctacaggtctctgaggatcatcttctc |
| mV1_600 | gtgacactgtaggaggatgggaagaataactcactg | cccatcctcctacagtgtcactacggctgtggaaaa |
| mV1_603 | atcgaggattagaagaataactcactgcctgtggag | gttattcttctaatcctcgatcagtgtcactacggc |
| mV1_605 | gatgggaagtagaactcactgcctgtggagtcccca | cagtgagttctacttcccatcctcgatcagtgtcac |
| mV1_630 | tcttacaactagctgtattccacatgtctggagctg | ggaatacagctagttgtaagagttacctggcctgca |
| pV1_643 | tcctataactagctgtactctacttgcctggagctc | agagtacagctagttataggaaggggaagaaggctt |
| mV1_633 | agcctgtattagacatgtctggagctgttcaagttc | cagacatgtctaatacaggctgttgtaagagttacc |
| mV1_636 | tccacatgttaggagctgttcaagttcaccatcggc | gaacagctcctaacatgtggaatacaggctgttgta |
| mV1_640 | gagctgttctagttcaccatcggcatgggtgacctg | gatggtgaactagaacagctccagacatgtggaata |
| mV1_648 | atgggtgactaggagttcaccgagaactatgacttc | ggtgaactcctagtcacccatgccgatggtgaactt |
| mV1_650 | gacctggagtagaccgagaactatgacttcaaggct | gttctcggtctactccaggtcacccatgccgatggt |
| mV1_651 | ctggagttctaggagaactatgacttcaaggctgtc | atagttctcctagaactccaggtcacccatgccgat |
| mV1_654 | accgagaactaggacttcaaggctgtcttcatcatc | cttgaagtcctagttctcggtgaactccaggtcacc |
| mV1_656 | aactatgactagaaggctgtcttcatcatcctgtta | gacagccttctagtcatagttctcggtgaactccag |
| mV1_672 | attctcacctagatcctcctgctcaacatgctcatt | caggaggatctaggtgagaatcacataggccagtaa |
| mV1_673 | ctcacctactagctcctgctcaacatgctcattgct | gagcaggagctagtaggtgagaatcacataggccag |
| mV1_676 | atcctcctgtagaacatgctcattgctctcatgggc | gagcatgttctacaggaggatgtaggtgagaatcac |
| mV1_677 | ctcctgctctagatgctcattgctctcatgggcgag | aatgagcatctagagcaggaggatgtaggtgagaat |
| mV1_680 | aacatgctctaggctctcatgggcgagactgtcaac | catgagagcctagagcatgttgagcaggaggatgta |
| mV1_684 | gctctcatgtaggagactgtcaacaagattgcacaa | gacagtctcctacatgagagcaatgagcatgttgag |
| mV1_685 | ctcatgggctagactgtcaacaagattgcacaagag | gttgacagtctagcccatgagagcaatgagcatgtt |

## Supplementary Methods

## Commands in Rosetta to perform loop modeling:

```
/home/fanyang/rosetta/main/source/bin/loopmodel.linuxgccrelease \
-in:path:database /home/fanyang/rosetta2015.25/main/database \
-score:weights membrane_highres_Menv_smooth.wts \
-in:file:fullatom \
-membrane:normal_cycles 100 \
-membrane:normal_mag 15 \
-membrane:center_search \
-ignore_unrecognized_res \
-symmetry:symmetry_definition
/home/fanyang/projects/input_files/3J5Q_2018/4D_ABCD_r_4D_after_ccd_relaxed_r.s
ymm \
-symmetry:initialize_rigid_body_dofs \
-in:file:spanfile /home/fanyang/projects/input_files/3J5Q_2018/3J5Q_FL.span \
-in:file:s                    /share/work/fanyang/work/3J5Q_FL_KIC5-3J5Q_2018-
_/top20_score_filtered/_1D/${SLURM_ARRAY_TASK_ID}.pdb \
-loops:loop_file /home/fanyang/projects/input_files/3J5Q_2018/3J5Q_FL_SF.loop \
-loops:remodel perturb_kic \
-loops:refine refine_kic \
-loops:relax no \
-loops:strict_loops \
-loops:build_attempts 20 \
-relax:bb_move false \
-max_inner_cycles 30 \
-nstruct 51 \
-out:prefix msymm-loop-kic- \
-out:file:silent              /share/work/fanyang/work/3J5Q_FL_KIC6-3J5Q_2018-
_/${SLURM_ARRAY_TASK_ID}/msymm-relax-_3J5Q_2018_${SLURM_ARRAY_TASK_ID}.silent \
-out:file:silent_struct_type binary \
-mute all
```

**Rosetta scripts to perform SASA calculation and filtering:**

```
<ROSETTASCRIPTS>
    <RESIDUE_SELECTORS>
    <Not name="630_unselected">
        <Index resnums=272/>
    </Not>
    </RESIDUE_SELECTORS>
    <TASKOPERATIONS>
    <OperateOnResidueSubset name="630_only" selector="6300_unselected" >
        <PreventRepackingRLT/>
    </OperateOnResidueSubset>
    </TASKOPERATIONS>
    <SCOREFXNS>
    </SCOREFXNS>
 <FILTERS>
    <TotalSasa     name=630_sasa     threshold=10     task_operations=630_only
report_per_residue_sasa=1/>
 </FILTERS>
    <MOVERS>
    </MOVERS>
    <PROTOCOLS>
    <Add filter=630_sasa/>
    </PROTOCOLS>

</ROSETTASCRIPTS>
```

**Commands in Rosetta to incorporate ANAP into TRPV1 models**

```
-database /home/fan/rosetta/main/database
-in:file:fullatom
-ignore_unrecognized_res
-s /home/fan/Rosetta/Project_ANAP_Parameterization/3J5Q.pdb
-backrub:ntrials 10
-nstruct 1
-mc_kt 0.6
-resfile /home/fan/Rosetta/Project_ANAP_Parameterization/630ANP_3J5Q.resfile
-overwrite
```

630ANP_3J5Q.resfile:

```
NATRO
start
630 A EMPTY NC ANP
630 B EMPTY NC ANP
630 C EMPTY NC ANP
630 D EMPTY NC ANP
```
