# Supplementary material for: Molecular basis for heat desensitization of TRPV1 ion channels
Source: Nat Commun. 2019 May 13;10:2134. doi: 10.1038/s41467-019-09965-6 (PMC6513986; doi:10.1038/s41467-019-09965-6)
Supplement: Supplementary file 3 — Reporting Summary [file 41467_2019_9965_MOESM3_ESM.pdf]

nature research

Corresponding author(s): Jie Zheng
NCOMMS-18-35115A

Last updated by author(s): Mar 20, 2019

Reporting Summary

Nature Research wishes to improve the reproducibility of the work that we publish. This form provides structure for consistency and transparency in reporting. For further information on Nature Research policies, see Authors & Referees and the Editorial Policy Checklist.

Statistics

For all statistical analyses, confirm that the following items are present in the figure legend, table legend, main text, or Methods section.

n/a	Confirmed	
☐	☒	The exact sample size (n) for each experimental group/condition, given as a discrete number and unit of measurement
☐	☒	A statement on whether measurements were taken from distinct samples or whether the same sample was measured repeatedly
☐	☒	The statistical test(s) used AND whether they are one- or two-sided<br>*Only common tests should be described solely by name; describe more complex techniques in the Methods section.*
☒	☐	A description of all covariates tested
☐	☒	A description of any assumptions or corrections, such as tests of normality and adjustment for multiple comparisons
☐	☒	A full description of the statistical parameters including central tendency (e.g. means) or other basic estimates (e.g. regression coefficient) AND variation (e.g. standard deviation) or associated estimates of uncertainty (e.g. confidence intervals)
☐	☒	For null hypothesis testing, the test statistic (e.g. F, t, r) with confidence intervals, effect sizes, degrees of freedom and P value noted<br>*Give P values as exact values whenever suitable.*
☒	☐	For Bayesian analysis, information on the choice of priors and Markov chain Monte Carlo settings
☒	☐	For hierarchical and complex designs, identification of the appropriate level for tests and full reporting of outcomes
☒	☐	Estimates of effect sizes (e.g. Cohen's d, Pearson's r), indicating how they were calculated

Our web collection on statistics for biologists contains articles on many of the points above.

Software and code

Policy information about availability of computer code

Data collection	Meta Image Series 7.8; PatchMaster v2x53; BIAcore 3000 Control Software version 4.1
Data analysis	Igor Pro 6.04; Graphad Prism 6; Office Excel

For manuscripts utilizing custom algorithms or software that are central to the research but not yet described in published literature, software must be made available to editors/reviewers. We strongly encourage code deposition in a community repository (e.g. GitHub). See the Nature Research guidelines for submitting code & software for further information.

Data

Policy information about availability of data

All manuscripts must include a data availability statement. This statement should provide the following information, where applicable:
- Accession codes, unique identifiers, or web links for publicly available datasets
- A list of figures that have associated raw data
- A description of any restrictions on data availability

All data supporting the findings in this study are available from the corresponding authors upon reasonable request.

Field-specific reporting

Please select the one below that is the best fit for your research. If you are not sure, read the appropriate sections before making your selection.

☒ Life sciences ☐ Behavioural & social sciences ☐ Ecological, evolutionary & environmental sciences

For a reference copy of the document with all sections, see nature.com/documents/nr-reporting-summary-flat.pdf

Life sciences study design

All studies must disclose on these points even when the disclosure is negative.

Sample size	Experiments were performed at least three independent times. Sample size in all experiments were estimated based in previous published experiments. At least three or more mice were used in animal experiment to obtain statistical analysis. In this study, the statistic analysis was obtained using two-sided t-test and the values represents means plus minus standard error of the mean.
Data exclusions	No data were excluded.
Replication	Since experiments were performed at least three independent times, all experimental findings were reproducible.
Randomization	Randomly selected samples and organisms were allocated into experimental groups.
Blinding	Since results and phenotypes of mice were so obvious, the blinding was unnecessary.

Reporting for specific materials, systems and methods

We require information from authors about some types of materials, experimental systems and methods used in many studies. Here, indicate whether each material, system or method listed is relevant to your study. If you are not sure if a list item applies to your research, read the appropriate section before selecting a response.

Materials & experimental systems

n/a	Involved in the study	
☐	☒	Antibodies
☐	☒	Eukaryotic cell lines
☒	☐	Palaeontology
☐	☒	Animals and other organisms
☒	☐	Human research participants
☒	☐	Clinical data

Methods

n/a	Involved in the study	
☒	☐	ChIP-seq
☒	☐	Flow cytometry
☒	☐	MRI-based neuroimaging

Antibodies

Antibodies used	Mouse β-Actin Antibody Santa sc-69879;<br>Caspase-3 (8G10) Rabbit mAb Cell Signaling Technology(CST) #9665.
Validation	Every antibodies used in this study was validated by their own company.

Eukaryotic cell lines

Policy information about cell lines

Cell line source(s)	HEK293T cells were purchased from Kunming Cell Bank, Kunming Institute of Zoology, Chinese Academy of Sciences (ATCC, CRL-3216).
Authentication	None of the cell line used were authenticated.
Mycoplasma contamination	Negative.
Commonly misidentified lines<br>(See ICLAC register)	The study does not use misidentified lines.

Animals and other organisms

Policy information about studies involving animals; ARRIVE guidelines recommended for reporting animal research

Laboratory animals	Platypus trpv1 knock-in mice (p-trpv1 mice) in the C57BL/6L background were generated. C57BL/6L mice were used as wild type mice. Both sexes were used in this study.
Wild animals	Wild animals were not involved in this study.
Field-collected samples	No samples were collected from field.
Ethics oversight	All the animal experiments were carried out in strict accordance with recommendations in the Guide for the Care and Use of

Ethics oversight	Laboratory Animals of Kunming Institute of Zoology, Chinese Academy of Sciences. Protocols were approved by the Institutional Animal Care and Use Committees at Kunming Institute of Zoology, Chinese Academy of Sciences (approval ID: SMKX2016023). All possible efforts were employed to reduce the number of animals used and also to minimize animal suffering.

Note that full information on the approval of the study protocol must also be provided in the manuscript.
